# Association of *Helicobacter pylori* infection with the risk of neurodegenerative disorders: a systematic review and meta-analysis

**DOI:** 10.3389/fmed.2025.1573299

**Published:** 2025-07-04

**Authors:** Jiwen Du, Weiting Shen, Zhenting Zhou, Qiuyan Wu, Zongyao Ai

**Affiliations:** ^1^Department of Gastroenterology, Wuxing District People’s Hospital, Wuxing District Maternal and Child Health Hospital Huzhou City, Zhejiang, China; ^2^Department of Pneumology, Wuxing District People’s Hospital, Wuxing District Maternal and Child Health Hospital Huzhou City, Zhejiang, China; ^3^Department of Neurology, Huzhou Hospital of Traditional Chinese Medicine Affiliated to Zhejiang University of Chinese Medicine, Huzhou, China

**Keywords:** *Helicobacter pylori* infection, Parkinson’s disease, all-cause dementia, Alzheimer’s disease, multiple sclerosis, meta-analysis

## Abstract

**Objective:**

The pathogenesis of neurodegenerative diseases is complex, involving multiple factors, including genetic, environmental, and lifestyle elements. Recent studies have suggested that infectious agents may act as important triggers for neurodegenerative diseases. This study aimed to evaluate the association between *Helicobacter pylori* (*H. pylori*) infection and the risk of neurodegenerative disorders through a systematic review and meta-analysis of existing literature.

**Methods:**

A systematic search was conducted across the PubMed, Embase, and Cochrane Library databases for studies published up to December 2024. The combined effect sizes were expressed as odds ratios (OR) with 95% confidence intervals (CI) and were calculated using a random-effects model. Further exploratory analyses included sensitivity analyses, subgroup analyses, and assessment of publication bias.

**Results:**

Forty-one studies involving 159,220 participants were selected for the meta-analysis. We found that *H. pylori* infection was associated with an increased risk of Parkinson’s disease (OR: 1.70; 95% CI: 1.36–2.13; *p* < 0.001), all-cause dementia (OR: 1.56; 95% CI: 1.24–1.96; *p* < 0.001), and Alzheimer’s disease (OR: 1.43; 95% CI: 1.01–2.02; *p* = 0.045). However, *H. pylori* infection was not associated with the risk of multiple sclerosis (OR: 0.81; 95% CI: 0.59–1.11; *p* = 0.193). Sensitivity analysis suggested that *H. pylori* infection might play a protective role in the subsequent risk of multiple sclerosis. Subgroup analyses indicated that the association between *H. pylori* infection and neurodegenerative disorders may vary based on country, study design, *H. pylori* detection technique, and study quality.

**Conclusion:**

This study found that *H. pylori* infection may be associated with an elevated risk of Parkinson’s disease, all-cause dementia, and Alzheimer’s disease.

**Systematic review registration:**

INPLASY (INPLASY202510044).

## Introduction

Neurodegenerative diseases constitute a heterogeneous set of chronic central nervous system maladies that are marked by progressive neuronal degeneration (1). These disorders exact a substantial toll on society, as they impact millions of people worldwide (2). Given the current lack of a cure and the fatality of some cases, patients necessitate long-term, uninterrupted care. This not only incurs substantial economic expenses but also exerts a heavy burden on social resources. It is estimated that by 2030, the global cost associated with dementia alone will surpass $2 trillion (3). With the global population aging and life expectancy rising, early diagnosis and intervention for neurodegenerative diseases have emerged as pressing public health imperatives.

Although research has indicated that elements like oxidative stress, viral infections, and the natural aging process play a role in the onset of these diseases (4–6), there is still a necessity to delve deeper into their potential risk factors. Pinpointing these risk factors can assist in identifying high-risk groups and enhancing patients’ prognoses and quality of life through early detection. Based on the infection hypothesis associated with neurodegenerative diseases, microorganisms might serve as initiators and/or mediators of these ailments (7). *Helicobacter pylori* (*H. pylori*) infection is a prevalent chronic infection that impacts more than 50% of the global population (8). In developing nations, the infection rate is estimated to be between 80 and 90%, whereas in the United States and Europe, it lies between 35 and 40% (9, 10). Research over the past decade has shown that chronic *H. pylori* infection is associated not only with various extraintestinal manifestations, such as atherosclerosis, hypertension, and stroke (11–13), but also with neurocognitive and neuropsychiatric disorders, including all-cause dementia and Alzheimer’s disease (AD) (14–16). *H. pylori* infection may contribute to these conditions by affecting the brain and vascular systems. These diseases may be triggered by *H. pylori* infection through mechanisms such as reduced nutrient bioavailability, alterations in gut microbiota, oxidative stress, and metabolic disturbances (17, 18). *H. pylori* infection may also lead to pathological changes resembling those observed in AD. Consequently, preventing chronic *H. pylori* infection could have a significant impact on the onset and progression of neurodegenerative diseases.

Recent research has extensively discussed the role of inflammation induced by *H. pylori* in the pathogenesis of Parkinson’s disease (PD) and AD (19). Additionally, another important article has pointed out that while there is growing interest in the relationship between Helicobacter species and neurological disorders, data on the association with multiple sclerosis (MS) remain limited (20). Previous meta-analyses have also explored the relationship between *H. pylori* infection and neurodegenerative diseases (21–23). Although these studies found *H. pylori* infection were associated with an increased risk of PD, and all-cause dementia, whereas it did not affect subsequent AD risk (21, 22). Moreover, the study conducted by Yao et al. suggested *H. pylori* infection was associated with a reduced risk of MS (23). However, there are still some inconsistencies in the results of different meta-analyses. These discrepancies may be due to differences in study populations, inclusion criteria, and the quality of the original studies included in the meta-analyses. Understanding these previous findings is crucial for our current study, as it allows us to build on existing knowledge and further explore the complex relationship between *H. pylori* infection and neurodegenerative diseases such as PD, all-cause dementia, AD, and MS.

## Materials and methods

### Data sources, search strategy, and selection criteria

This review was carefully designed and reported in accordance with the 2020 updated Preferred Reporting Items for Systematic Reviews and Meta-Analyses (PRISMA) statement to ensure the transparency and reliability of the research process (PRISMA Checklist) (24). Our study was registered in INPLASY platform (number: INPLASY202510044). The objective was to comprehensively evaluate existing evidence on the potential association between *H. pylori* infection and neurodegenerative diseases. To maximize the dataset, no restrictions were placed on the language or publication status of the included studies.

A systematic search was conducted in PubMed, Embase, and the Cochrane Library for relevant articles published up to December 2024, using the search terms “*Helicobacter pylori*” AND “neurocognitive disorders.” The detailed search strategy for PubMed is provided in Supplementary Table S1. Additionally, a manual search was performed by reviewing the reference lists of all relevant primary studies and review articles to identify studies that may not have been indexed in the databases but still met the inclusion criteria. This approach helped minimize the risk of missing important studies.

Study relevance was determined by evaluating medical subject headings, research methods, patient populations, study designs, exposure factors, and outcome variables. The literature search was independently conducted by two authors using a standardized method. Any discrepancies that arose during the screening process were resolved through discussion, and if consensus could not be reached, the principal author made the final decision.

Studies were included if they met the following criteria: they used either a case–control or cohort study design, evaluated the association between *H. pylori* infection and neurodegenerative diseases, and reported effect estimates with corresponding 95% confidence intervals (95% CI). To ensure accuracy and consistency, the two authors independently reviewed the titles and abstracts of each article to preliminarily select studies that met the inclusion criteria. These selected studies then underwent a full-text review to confirm their compliance with all inclusion criteria. Cross-sectional studies were excluded because they could not establish a causal relationship.

### Data collection and quality assessment

Data collected from each study included the first author’s name, publication year, country, study design, sample size, baseline age, percentage of male participants, definition of *H. pylori* infection, reported endpoints, effect estimates with their 95% confidence intervals, and details of the adjusted models. When dealing with studies that reported outcomes for both all-cause dementia and AD, we implemented a systematic approach to avoid double-counting. For the studies that reported data for both endpoints, we extracted the data separately based on the specific outcome of interest for each meta-analysis. In the meta-analysis of all-cause dementia, we only included the data related to the all-cause dementia outcome from these studies. Similarly, for the meta-analysis of AD, we exclusively used the data specifically reported for AD. This method ensured that each data point was utilized only once, depending on the relevant outcome, and prevented any over- or under-estimation of the associations. The methodological quality of the included studies was assessed using the Newcastle-Ottawa Scale (NOS), a widely validated tool for evaluating observational studies. The NOS consists of three sections: Selection (4 items), Comparability (1 item), and Outcome (3 items), with a total score ranging from 0 to 9. Higher scores indicate better study quality (25). Data extraction and quality assessment were independently conducted by two authors to ensure objectivity and consistency. Both authors thoroughly reviewed each study, extracted the required data, and assessed study quality using the NOS. A third author independently checked the original studies to verify data accuracy and completeness and resolved any discrepancies if necessary.

### Statistical analysis

The association between *H. pylori* infection and the risk of PD, all-cause dementia, AD, and MS was assessed based on the effect estimates and their 95% confidence intervals reported in each study. A random-effects model was used to pool the odds ratios (ORs) and 95% confidence intervals to account for heterogeneity across studies (26, 27). Heterogeneity among the studies was evaluated using the *I^2^* statistic and Q test. An *I^2^* value greater than 50.0% or a *p*-value less than 0.10 was considered indicative of significant heterogeneity (28, 29). Sensitivity analyses were conducted to assess the robustness of the pooled results (30). Subgroup analyses were carried out based on country, study design, *H. pylori* detection methods, reported outcomes, and study quality. Differences between subgroups were compared using the interaction t-test, assuming that the data followed a normal distribution (31). In addition to the subgroup analyses, we performed meta-regression analyses to further explore the sources of heterogeneity (32). Publication bias was visually assessed through funnel plots for PD, all-cause dementia, AD, and MS. Additionally, Egger’s and Begg’s tests were performed to statistically evaluate publication bias (33, 34). All reported *p*-values were two-sided, and a *p*-value of less than 0.05 was considered statistically significant. All statistical analyses were conducted using STATA software (version 12.0; StataCorp, College Station, TX, USA).

## Results

### Literature search

**Figure 1:**
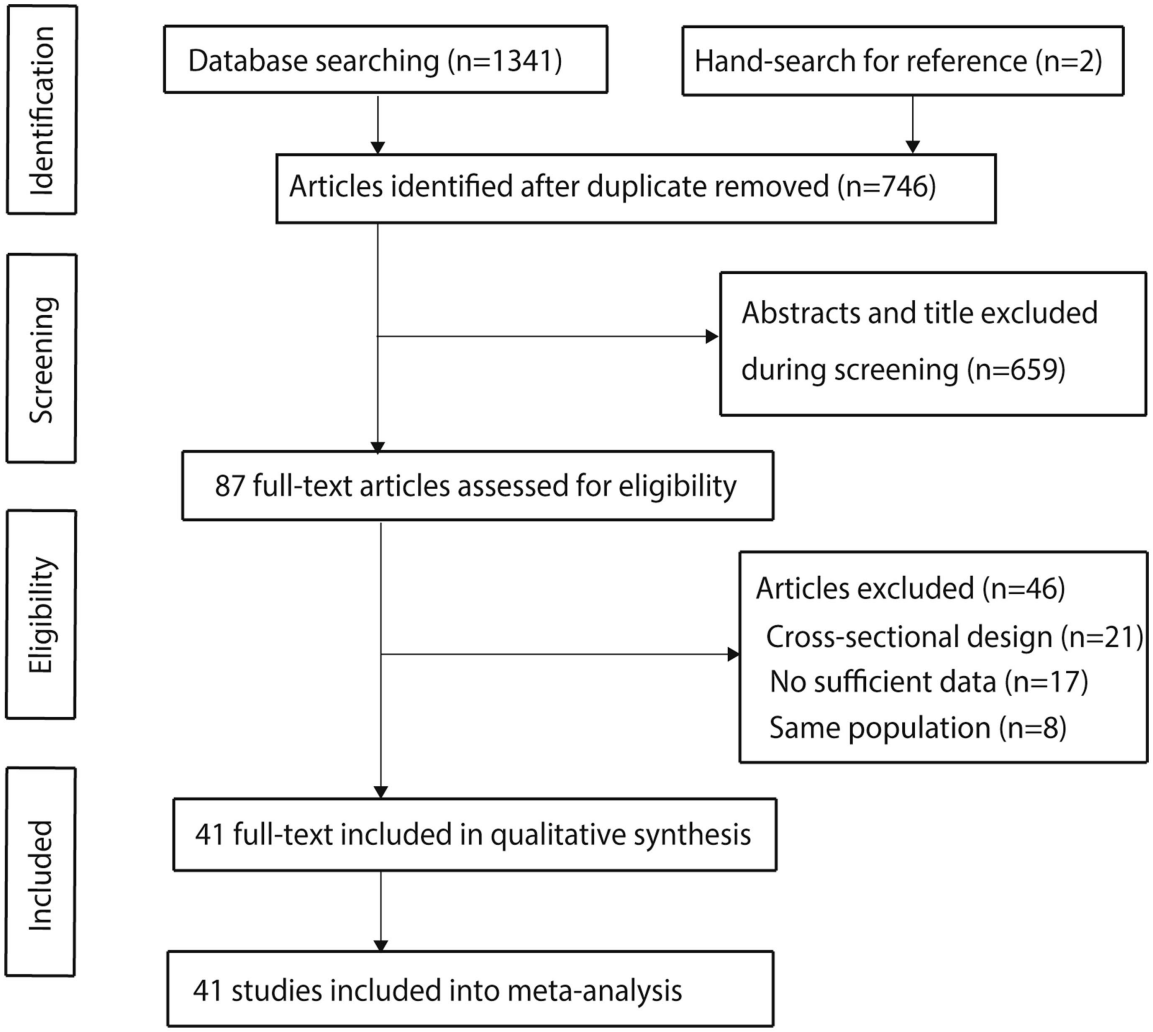
Flow diagram of the literature search and study selection process.

The detailed screening process for the included studies is presented in Figure 1. Initially, 1,341 articles were identified through electronic searches. After removing duplicates, 746 articles remained. Following the screening of titles and abstracts, 659 articles were excluded. The remaining 87 studies underwent a full-text review, resulting in 41 studies that met the inclusion criteria and were included in the meta-analysis (35–75). A manual search of reference lists from these studies identified two additional studies that initially appeared to meet the criteria; however, further screening revealed that these studies were already included in the electronic search results and were therefore excluded.

### Study characteristics

Table 1 summarizes the baseline characteristics of the included studies and their participants. Collectively, these studies involved 159,220 participants. Of these, 11 studies employed a cohort design, while the remaining 30 studies used a case–control design. Seventeen studies were conducted in Eastern countries, mainly China, Japan, Iran, India, and Singapore, while the other 24 studies were conducted in Western countries, primarily in Europe and North America. Eighteen studies reported adjusted effect estimates, whereas 23 studies provided unadjusted effect estimates. The quality of the studies, assessed using the NOS, showed that six studies scored 8, twelve studies scored 7, eleven studies scored 6, and the remaining twelve studies scored 5. The inclusion of these lower-quality studies may potentially introduce biases into the meta-analysis results, which will be further discussed in the subsequent sections.

**Table 1:** The baseline characteristics of included studies and involved participants.

Study	Country	Study design	Sample size	Age (years)	Male (%)	*H. pylori* infection detection	Reported outcomes	Adjusted	Study quality
Charlett 1999 (35)	UK	Case-control	111	70.5	41.0	ELISA	PD	Adjusted	6
Dobbs 2000 (36)	UK	Cohort	315	67.5	51.4	ELISA	PD	Adjusted	7
Nägga 2003 (37)	Sweden	Case-control	224	71.9	44.2	ELISA	AD, VaD	Adjusted	5
Kountouras 2006 (38)	Greece	Case-control	80	64.0	40.0	ELISA	AD	Crude	5
Li 2007 (39)	Japan	Case-control	190	42.2	23.7	ELISA	MS	Crude	6
Kountouras 2007 (40)	Greece	Case-control	98	67.1	39.8	Biopsy and ELISA	MCI	Crude	6
Li 2009 (41)	Japan	Case-control	247	39.7	25.1	ELISA	MS	Crude	6
Charlett 2009 (42)	UK	Case-control	586	59.5	50.3	ELISA	PD	Crude	7
Kountouras 2009 (43)	Greece	Case-control	80	65.0	40.0	Biopsy and ELISA	AD	Crude	5
Zarkesh 2009 (44)	Iran	Case-control	399	NA	32.9	ELISA	MS	Crude	5
Riskind 2010 (45)	USA	Case-control	410	NA	NA	Latex agglutination	MS	Crude	6
Shiota 2011 (46)	Japan	Case-control	482	75.2	27.2	ELISA	AD	Adjusted	7
Nielsen 2012 (47)	Denmark	Case-control	26,900	70.8	59.7	ICD-8 and 10	PD	Adjusted	8
Ramroodi 2012 (48)	Iran	Case-control	201	NA	NA	Western blot	MS	Crude	5
Mohebi 2013 (49)	Iran	Case-control	313	32.0	54.0	ELISA	MS	Crude	6
Yoshimura 2013 (50)	Japan	Case-control	299	31.4	35.2	ELISA	MS	Crude	5
Long 2013 (51)	China	Case-control	69	33.9	50.0	IF	MS	Adjusted	7
Ram 2013 (52)	Latin America	Case-control	238	NA	NA	ICT	MS	Crude	5
Blaecher 2013 (53)	UK	Case-control	316	52.0	48.7	Biopsy and ELISA	PD	Crude	6
Baudron 2013 (54)	France	Cohort	603	73.9	42.3	ELISA	All-cause dementia	Adjusted	8
Nafisah 2013 (55)	USA	Case-control	52	64.1	NA	ELISA	PD	Crude	5
Huang 2014 (56)	China	Cohort	83,965	63.3	61.6	ICD-9	All-cause dementia, AD	Adjusted	7
Cook 2015 (57)	UK	Case-control	113	51.9	31.0	ELISA	MS	Crude	5
Pedrini 2015 (58)	Australia	Case-control	849	35.4	25.8	ELISA	MS	Crude	6
Malli 2015 (59)	India	Case-control	417	36.7	33.8	ELISA	MS	Adjusted	7
Gavalas 2015 (60)	Greece	Case-control	64	36.7	28.1	Biopsy	MS	Crude	5
Bu 2015 (61)	China	Case-control	272	67.5	52.6	ELISA	PD	Adjusted	7
Tsolaki 2015 (62)	Greece	Case-control	156	62.1	44.2	Biopsy	PD, AD	Crude	5
Bu 2015 (63)	China	Case-control	263	69.5	49.8	ELISA	AD	Adjusted	7
Deretzi 2016 (64)	Greece	Case-control	68	29.7	22.9	ELISA	MS	Crude	6
Efthymiou 2017 (65)	Greece	Case-control	267	50.7	36.0	ELISA	MS, PD, AD	Crude	5
Fani 2018 (66)	Netherlands	Cohort	4,215	68.4	45.7	ELISA	All-cause dementia, AD	Adjusted	8
Huang 2018 (67)	China	Cohort	18,210	50.9	53.3	ICD-9	PD	Adjusted	7
Ranjbar 2019 (68)	Iran	Case-control	806	31.0	48.2	ELISA	MS	Crude	6
Kiani 2021 (69)	Iran	Case-control	183	35.8	16.8	ELISA	MS	Crude	6
Zilli 2021 (70)	USA	Cohort	6,468	66.5	44.3	ELISA	All-cause dementia	Adjusted	8
Fu 2023 (71)	UK	Cohort	8,144	56.5	43.7	ICD-9	All-cause dementia, AD	Crude	8
Wang 2023 (72)	China	Cohort	268	70.9	46.6	14\u00B0C urea breath test	MCI	Adjusted	7
Davis 2024 (73)	Australia	Cohort	1,115	64.0	48.0	ELISA	All-cause dementia	Adjusted	7
Hernandez-Ruiz 2024 (74)	France	Cohort	689	75.8	62.0	ELISA	All-cause dementia	Adjusted	7
Shi 2024 (75)	Singapore	Cohort	475	67.6	41.3	ELISA	All-cause dementia	Adjusted	8

### Parkinson’s disease

**Figure 2:**
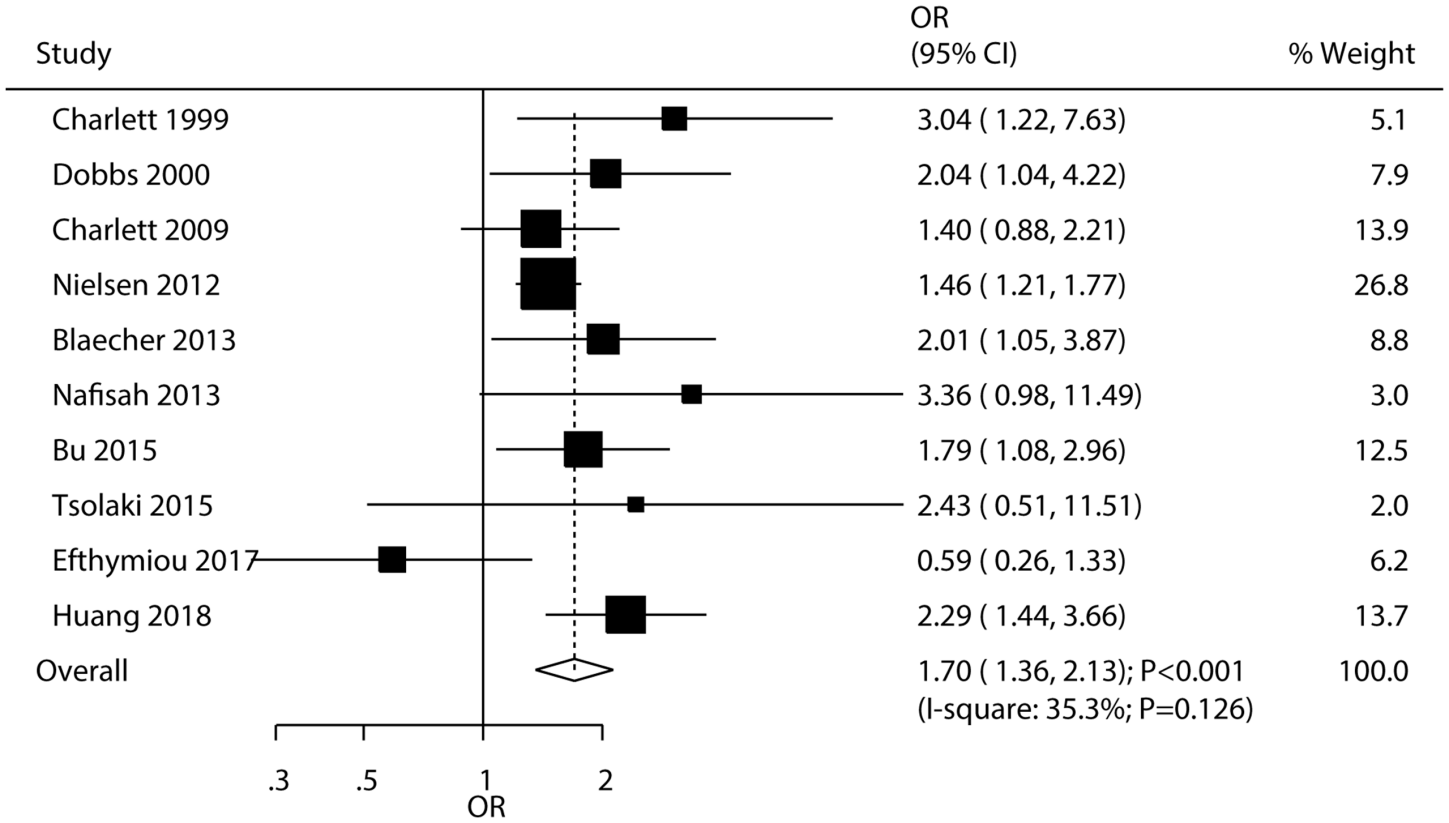
Association between *Helicobacter pylori* (*H. pylori*) infection and the risk of Parkinson’s disease (PD).

A total of 10 studies evaluated the association between *H. pylori* infection and the risk of PD. The meta-analysis indicated that *H. pylori* infection was associated with an increased risk of PD (OR: 1.70; 95% CI: 1.36–2.13; *p* < 0.001; Figure 2), with low heterogeneity observed among the studies (*I^2^* = 35.3%; *p* = 0.126). Sensitivity analysis demonstrated that the pooled conclusion was robust and remained unchanged when any single study was sequentially removed (Supplementary Figure S1). Subgroup analyses showed that *H. pylori* infection was associated with an increased risk of PD in most subsets. However, no significant association was observed in studies reporting crude data or those with low quality (Table 2). Meta-regression analyses found the mean age (*p* = 0.660) and male proportion (*p* = 0.592) were not significant factors contributing to the association between *H. pylori* infection and PD (Supplementary Figures S2, S3). There was no significant publication bias concerning the association between *H. pylori* infection and the risk of PD (Egger’s test: *p* = 0.272; Begg’s test: *p* = 0.474; Supplementary Figure S4).

**Table 2:** Subgroup analyses for neurodegenerative disorders.

Outcome	Factors	Subgroups	No of studies	OR and 95%CI	*p* value	*I^2^* (%)	Q statistic	Interaction test
PD	Country	Eastern	2	2.04 (1.45–2.88)	< 0.001	0.0	0.482	0.119
		Western	8	1.61 (1.22–2.12)	0.001	36.3	0.139	
	Study design	Case-control	8	1.59 (1.23–2.05)	< 0.001	34.6	0.152	0.077
		Cohort	2	2.21 (1.50–3.26)	< 0.001	0.0	0.788	
	Hp detection	ELISA	7	1.70 (1.20–2.39)	0.003	42.2	0.109	0.706
		Other	3	1.73 (1.22–2.46)	0.002	40.8	0.185	
	Outcome	Adjusted	5	1.78 (1.40–2.27)	< 0.001	29.0	0.228	0.523
		Crude	5	1.50 (0.90–2.51)	0.120	49.1	0.097	
	Study quality	High	5	1.59 (1.35–1.86)	< 0.001	2.6	0.392	0.685
		Low	5	1.83 (0.94–3.58)	0.076	58.5	0.047	
All-cause dementia	Country	Eastern	3	1.95 (1.29–2.95)	0.002	55.2	0.107	0.002
		Western	9	1.45 (1.11–1.89)	0.007	76.2	< 0.001	
	Study design	Case–control	3	3.97 (0.81–19.49)	0.090	90.0	< 0.001	0.034
		Cohort	9	1.37 (1.14–1.65)	0.001	65.0	0.004	
	Hp detection	ELISA	9	1.60 (1.18–2.17)	0.002	79.2	< 0.001	0.039
		Other	3	1.54 (1.11–2.13)	0.009	55.8	0.104	
	Outcome	Adjusted	9	1.37 (1.13–1.66)	0.001	65.7	0.003	0.068
		Crude	3	4.10 (0.88–19.08)	0.072	90.3	< 0.001	
	Study quality	High	9	1.37 (1.14–1.65)	0.001	65.0	0.004	0.034
		Low	3	3.97 (0.81–19.49)	0.090	90.0	< 0.001	
AD	Country	Eastern	3	1.01 (0.69–1.49)	0.941	19.9	0.287	0.305
		Western	6	1.83 (1.09–3.08)	0.023	77.5	< 0.001	
	Study design	Case-control	5	1.70 (0.92–3.14)	0.090	68.6	0.013	0.265
		Cohort	4	1.27 (0.79–2.06)	0.326	74.5	0.008	
	Hp detection	ELISA	6	1.54 (0.98–2.44)	0.064	75.8	0.001	0.914
		Other	3	1.25 (0.64–2.46)	0.509	61.0	0.077	
	Outcome	Adjusted	5	1.22 (0.83–1.80)	0.303	68.6	0.013	0.078
		Crude	4	2.04 (0.92–4.52)	0.077	69.7	0.019	
	Study quality	High	6	1.22 (0.89–1.68)	0.209	61.0	0.025	0.018
		Low	3	2.63 (0.84–8.18)	0.096	72.6	0.026	
MS	Country	Eastern	11	0.69 (0.50–0.96)	0.027	75.4	< 0.001	0.421
		Western	6	1.30 (0.56–3.04)	0.545	86.8	< 0.001	
	Study design	Case–control	17	0.81 (0.59–1.11)	0.193	79.8	< 0.001	–
		Cohort	0	–	–	–	–	
	Hp detection	ELISA	12	0.64 (0.46–0.89)	0.008	78.5	< 0.001	< 0.001
		Other	5	1.53 (0.89–2.61)	0.124	48.2	0.102	
	Outcome	Adjusted	2	0.77 (0.14–4.20)	0.759	88.5	0.003	0.278
		Crude	15	0.83 (0.59–1.15)	0.258	79.8	< 0.001	
	Study quality	High	2	0.77 (0.14–4.20)	0.759	88.5	0.003	0.278
		Low	15	0.83 (0.59–1.15)	0.258	79.8	< 0.001	

### All-cause dementia

**Figure 3:**
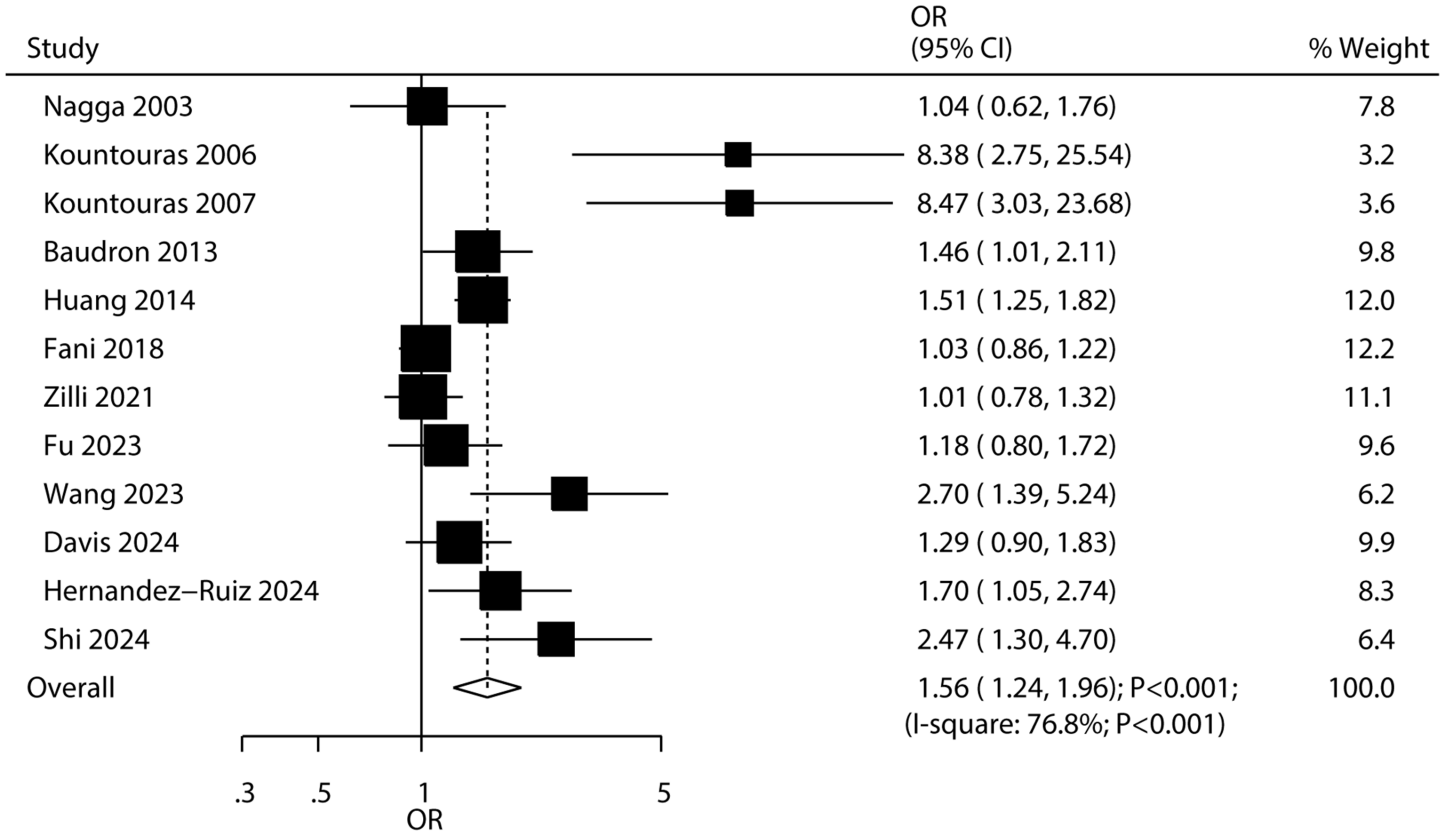
Association between *H. pylori* infection and the risk of dementia.

Twelve studies examined the association between *H. pylori* infection and the risk of all-cause dementia. The results indicated that *H. pylori* infection was associated with an increased risk of all-cause dementia (OR: 1.56; 95% CI: 1.24–1.96; *p* < 0.001; Figure 3), with significant heterogeneity detected among the included studies (*I^2^* = 76.8%; *p* < 0.001). Sensitivity analysis confirmed that the pooled conclusion was stable and was not altered by the exclusion of any particular study (Supplementary Figure S5). While the significant association persisted in most subgroup analyses, no association was observed when pooling case–control studies, studies reporting crude data, or studies with low quality (Table 2). Meta-regression analyses found the mean age (*p* = 0.954) and male proportion (*p* = 0.474) were not significant factors contributing to the association between *H. pylori* infection and all-cause dementia (Supplementary Figures S6, S7). Significant publication bias was detected for the association between *H. pylori* infection and the risk of all-cause dementia (Egger’s test: *p* = 0.009; Begg’s test: *p* = 0.007; Supplementary Figure S8). However, after adjusting for potential publication bias, the conclusion remained stable.

### Alzheimer’s disease

**Figure 4:**
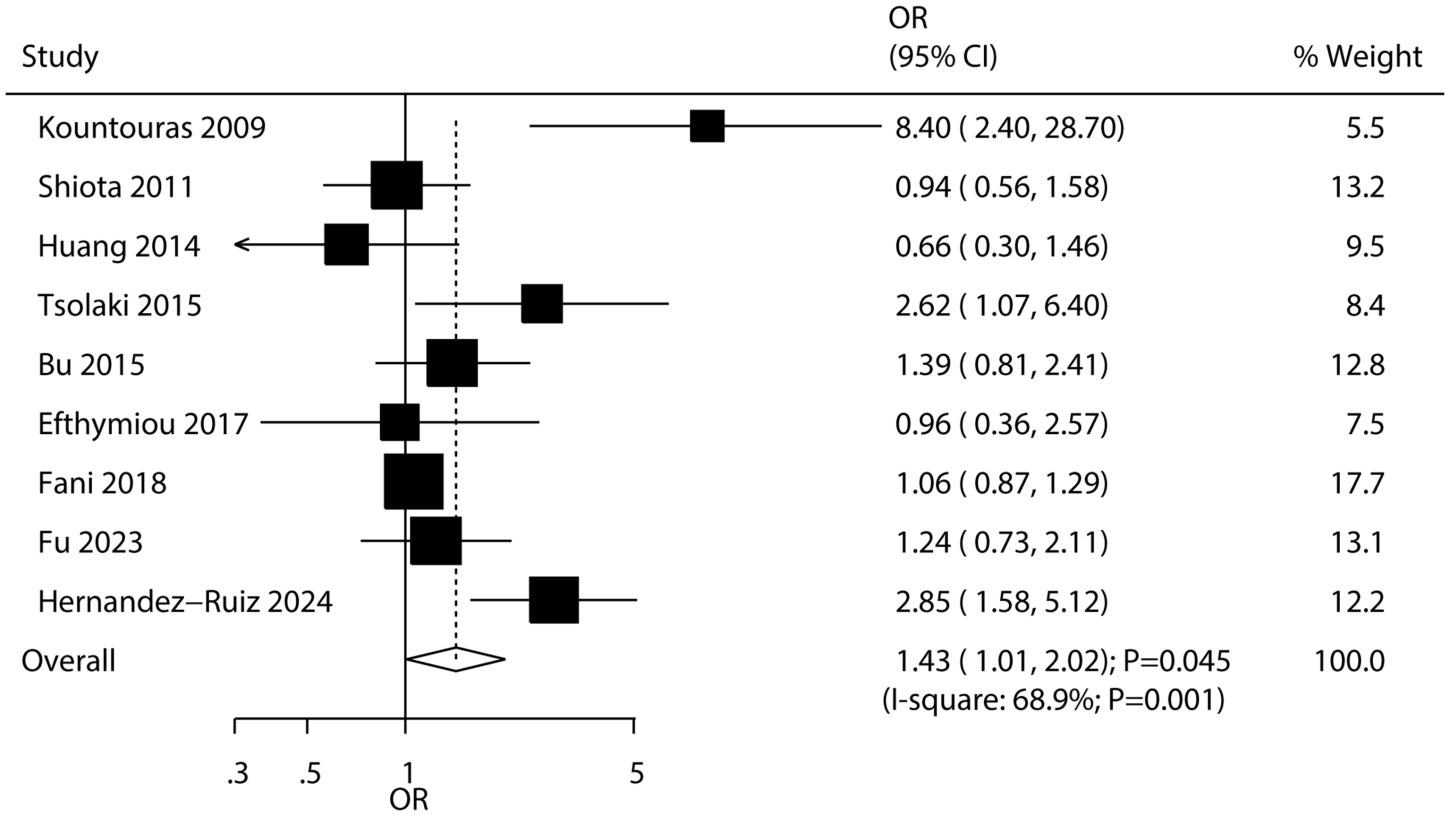
Association between *H. pylori* infection and the risk of Alzheimer’s disease (AD).

Nine studies explored the association between *H. pylori* infection and the risk of AD. The pooled results showed that *H. pylori* infection was associated with an increased risk of AD (OR: 1.43; 95% CI: 1.01–2.02; *p* = 0.045; Figure 4), with significant heterogeneity across studies (*I^2^* = 68.9%; *p* = 0.001). Sensitivity analysis suggested that the pooled conclusion was not stable due to the marginal confidence interval (Supplementary Figure S9). Subgroup analysis revealed that *H. pylori* infection was significantly associated with an increased risk of AD in studies conducted in Western countries (Table 2). Meta-regression analyses found the mean age (*p* = 0.687) and male proportion (*p* = 0.799) were not significant factors contributing to the association between *H. pylori* infection and AD (Supplementary Figures S10, S11). No significant publication bias was detected regarding this association (Egger’s test: *p* = 0.167; Begg’s test: *p* = 0.076; Supplementary Figure S12).

### Multiple sclerosis

**Figure 5:**
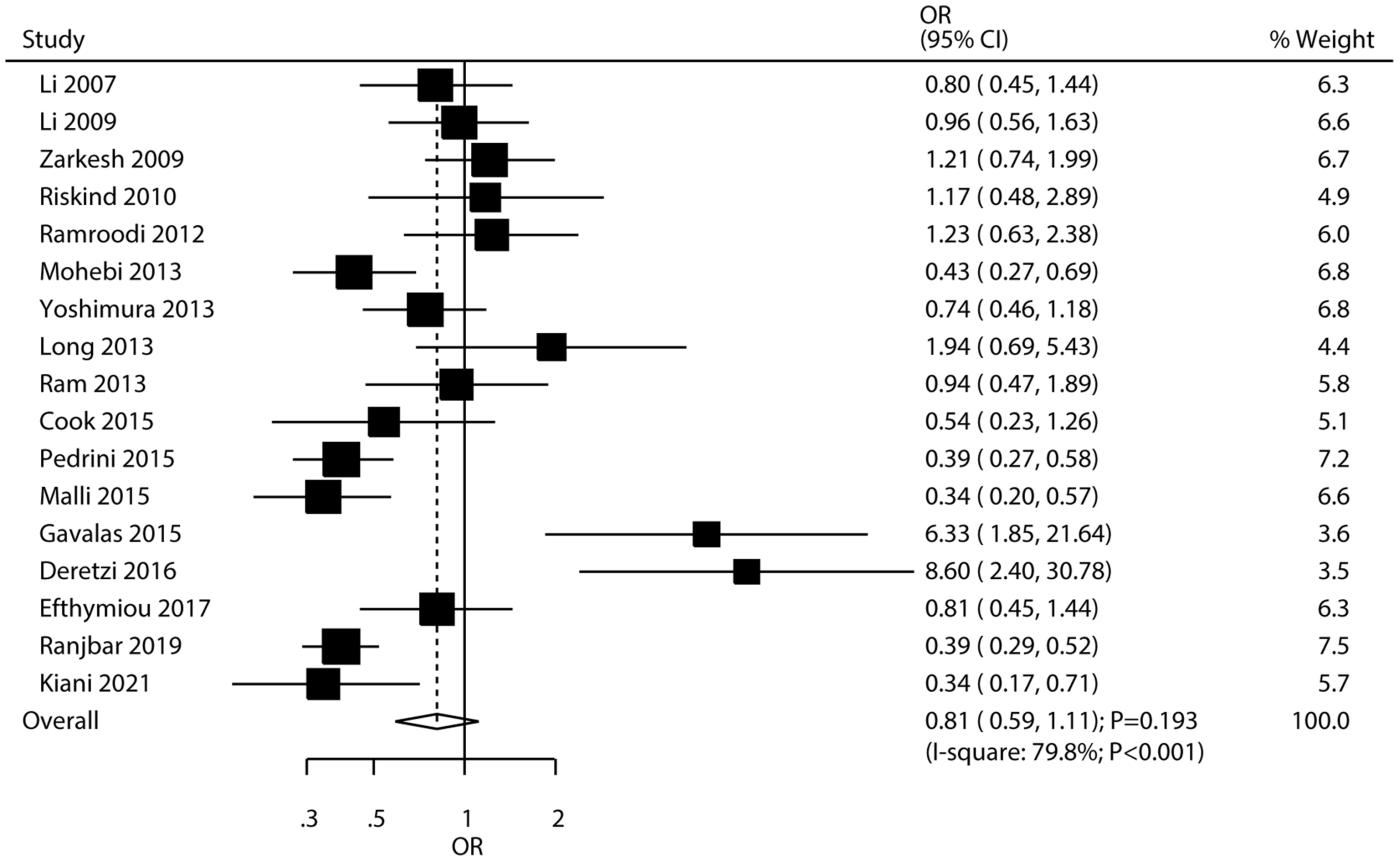
Association between *H. pylori* infection and the risk of multiple sclerosis (MS).

Seventeen studies investigated the association between *H. pylori* infection and the risk of MS. The meta-analysis found no significant association between *H. pylori* infection and the risk of MS (OR: 0.81; 95% CI: 0.59–1.11; *p* = 0.193; Figure 5), with significant heterogeneity among studies (*I^2^* = 79.8%; *p* < 0.001). However, sensitivity analysis, which involved sequentially removing individual studies, revealed a potential inverse association. Specifically, when certain studies were excluded, the pooled odds ratio suggested that *H. pylori* infection might be associated with a lower risk of MS (Supplementary Figure S13), leading to the hypothesis of a possible “protective role” of *H. pylori* against MS. Subgroup analyses indicated that *H. pylori* infection was associated with a reduced risk of MS in studies conducted in Eastern countries and in studies that used ELISA to detect *H. pylori* infection (Table 2). Meta-regression analyses found the mean age (*p* = 0.715) and male proportion (*p* = 0.586) were not significant factors contributing to the association between *H. pylori* infection and MS (Supplementary Figures S14, S15). Significant publication bias was detected for the association between *H. pylori* infection and MS risk (Egger’s test: *p* = 0.001; Begg’s test: *p* = 0.003; Supplementary Figure S16). However, after adjusting for potential publication bias, the overall conclusion remained unchanged.

## Discussion

This study, based on epidemiological research, comprehensively explores the possible associations between *H. pylori* infection and neurodegenerative diseases. This large-scale quantitative analysis includes 159,220 individuals from 11 cohort studies and 30 case–control studies, providing broad population representation. The results indicate a significant association between *H. pylori* infection and an increased risk of PD, all-cause dementia, and AD. However, the association between *H. pylori* infection and MS was not statistically significant. Sensitivity analysis, however, suggested a significant association between *H. pylori* infection and a lower risk of MS. Further subgroup analyses offered detailed insights into the risk of *H. pylori* infection and neurodegenerative diseases across specific populations.

Recent research has emphasized the significance of specific *H. pylori* virulence factors in its association with neurodegenerative diseases. The cytotoxin-associated gene A (CagA) is one of the most well-studied virulence factors. CagA-positive *H. pylori* strains have been shown to induce a more robust inflammatory response compared to CagA-negative strains (76). Once injected into host cells, CagA can disrupt cellular signaling pathways, leading to the activation of pro-inflammatory cytokines and the recruitment of immune cells (77). In the context of AD, CagA may contribute to tau pathology. A recent study demonstrated that CagA can interact with tau proteins, promoting their phosphorylation and aggregation, which are key pathological hallmarks of AD (78). For PD, the chronic inflammation triggered by CagA-positive *H. pylori* strains may exacerbate the oxidative stress and neuroinflammation associated with the disease, accelerating the degeneration of dopaminergic neurons (79). Microbiota dysbiosis, influenced by *H. pylori* infection, is another critical aspect that has gained increasing attention. *H. pylori* colonization of the gastric mucosa can lead to significant alterations in the gut microbiota composition. These changes can disrupt the normal balance of commensal bacteria, affecting the production of short-chain fatty acids and other metabolites with immunomodulatory and neuroprotective properties (80). Moreover, the dysbiotic microbiota may interact with the immune system in ways that promote the development of neurodegenerative diseases, potentially through the activation of pro-inflammatory pathways and the disruption of immune tolerance (81). Molecular mimicry is an emerging mechanism by which *H. pylori* may contribute to neurodegenerative diseases, particularly in the case of MS. The surface proteins of *H. pylori* may share sequence or structural similarities with proteins in the central nervous system, leading to an autoimmune response. When the immune system recognizes *H. pylori* antigens, it may also mistakenly target self-proteins in the brain, triggering the autoimmune attack characteristic of MS (69). In addition, *H. pylori*-induced vitamin B12 malabsorption is an important factor in the context of neurodegenerative diseases. *H. pylori* infection can cause atrophic gastritis, which reduces the production of intrinsic factor, essential for vitamin B12 absorption (82). Vitamin B12 deficiency has been linked to cognitive impairment, demyelination, and axonal damage, all of which are relevant to the development of PD and AD (83). Addressing *H. pylori*-related vitamin B12 malabsorption could potentially be a target for intervention strategies in these neurodegenerative diseases. Finally, the potential role of *H. pylori* eradication in modulating neurodegenerative risk is an area of growing interest (84).

This study identified a significant association between *H. pylori* infection and a higher risk of PD, aligning with previous research findings (85). However, our study with a larger sample size and more diverse study populations provides stronger evidence for this association. This may be attributed to our more comprehensive literature search that included studies from both Eastern and Western countries, capturing a broader range of environmental and genetic factors that could influence the relationship. *H. pylori* infection can cause chronic inflammation in the gastrointestinal tract, leading to systemic inflammatory responses. This persistent inflammation may impact the brain by activating the immune system and releasing inflammatory mediators, thereby promoting neuroinflammation and neurodegeneration (86). Additionally, *H. pylori* infection can impair the intestinal barrier, increasing gut permeability and allowing harmful substances and bacterial products to enter the bloodstream. This process triggers systemic inflammation and oxidative stress, which can adversely affect the brain and elevate the risk of PD (87). Moreover, *H. pylori* infection can disrupt the gut microbiota balance, impairing the gut-brain axis and negatively impacting neurological health (88). Finally, the observed increased risk of PD associated with *H. pylori* infection may be correlated with the duration of infection. It is possible that the longer the body is exposed to *H. pylori*-induced inflammation, the greater the damage to the nervous system (89). Additionally, although we did not consider the severity of *H. pylori* infection in our analysis, it is reasonable to hypothesize that a more severe infection may lead to a higher risk. This could be due to a more intense systemic inflammatory response, which may cause greater oxidative stress and damage to neurons in the brain.

We also found a significant association between *H. pylori* infection and a higher incidence of all-cause dementia, consistent with previous meta-analyses, although the effect size observed in this study was somewhat smaller (21). This difference may be due to variations in study designs, inclusion criteria, and the quality of the original studies included in each meta-analysis. Our study, by including a more recent and extensive range of studies, provides an updated perspective on this association. Additionally, we will discuss how our subgroup analyses based on different factors contribute to a more nuanced understanding compared to previous work. *H. pylori* infection can disrupt the gut microbiota, leading to the production of harmful metabolites. These metabolites may cross the blood–brain barrier and trigger neuroinflammatory processes (86). We hypothesize that certain bacterial metabolites produced as a result of *H. pylori*-induced gut microbiota dysbiosis may interact with amyloid-beta and tau proteins in the brain, accelerating the formation of plaques and tangles characteristic of AD. In the case of dementia, the systemic inflammation caused by *H. pylori* may also lead to cognitive decline by affecting neurotransmitter systems and neuronal plasticity. However, findings regarding the relationship between *H. pylori* infection and AD remain somewhat inconsistent. This inconsistency may be due to several factors: (1) the specific impact of *H. pylori* on AD could be influenced by uncontrolled variables, and differences in these variables across studies may lead to varying conclusions; and (2) variations in participant inclusion criteria and the incorporation of more recent literature could contribute to the discrepancies between our findings and those of previous studies (88). Finally, since all-cause dementia encompasses a wide range of etiologies, the association we observed may reflect the cumulative impact of *H. pylori* infection on various neurodegenerative and vascular processes that contribute to cognitive decline. This highlights the potential importance of considering *H. pylori* infection as a risk factor across different subtypes of dementia, and further research is needed to explore its specific role in individual dementia etiologies.

The statistically significant ORs for PD and all-cause dementia indicate a notable association between *H. pylori* infection and these neurodegenerative disorders. These findings may prompt clinicians to consider *H. pylori* screening and treatment in patients at risk of PD or all-cause dementia, especially in regions with a high prevalence of *H. pylori* infection. However, it is important to note that while these associations are significant, they should be considered alongside other well-established risk factors for PD and all-cause dementia, such as age, genetic predisposition, and lifestyle factors. From a public health perspective, targeting *H. pylori* infection could potentially be an additional strategy for reducing the incidence of PD and all-cause dementia. Future studies are needed to explore the cost-effectiveness of such an approach and to determine the optimal timing and method of *H. pylori* intervention to maximize the potential benefits in preventing these debilitating neurodegenerative diseases.

This study found no statistically significant association between *H. pylori* infection and the risk of MS, consistent with previous research (90). However, the sensitivity analysis presented an intriguing finding. By sequentially removing individual studies, we observed a potential inverse association, which led us to propose a possible “protective role” of *H. pylori* against MS. This hypothesis can be partly explained by the “hygiene hypothesis.” According to this theory, early exposure to microorganisms like *H. pylori* may contribute to the maturation and balance of the immune system (91). In the context of MS, an autoimmune disease characterized by the immune system’s attack on the central nervous system, *H. pylori* infection might help regulate the immune response. For instance, it could activate regulatory T cells, reducing the overactive immune response that is characteristic of MS. Additionally, *H. pylori*-induced alterations in the gut microbiota may influence immune function in a way that decreases the likelihood of MS development. However, it is crucial to note that this “protective role” is based on sensitivity analysis results and remains speculative until further research can confirm the association.

Further exploratory analysis suggests that the association between *H. pylori* infection and neurodegenerative diseases may be influenced by factors such as country, study design, detection techniques, and study quality. Differences in environmental conditions, lifestyles, and healthcare systems across countries could lead to variations in the prevalence of *H. pylori* infection and its health impacts. Variations in study designs reflect challenges in establishing causal relationships and increase the potential for bias. Additionally, differences in detection methods for *H. pylori* infection affect diagnostic accuracy and contribute to inconsistencies in results. As demonstrated by the subgroup analyses, these methodological differences can have a substantial impact on the observed associations between *H. pylori* infection and neurodegenerative diseases. The inconsistent accuracy, sensitivity, and specificity of different detection methods, along with potential variations in how they are implemented across studies, can lead to misclassification of *H. pylori* infection status. This misclassification can either mask true associations or create spurious ones, as seen in the subgroup analysis for MS where the use of ELISA in Eastern studies suggested a protective effect. These findings highlight the need for more standardized and validated *H. pylori* detection methods in future research on the relationship between *H. pylori* infection and neurodegenerative diseases. Study quality, including sample size, data collection methods, and statistical analysis rigor, also plays a crucial role in the consistency and reliability of findings. Moreover, the presence of 12 studies with a NOS score of ≤5 in our meta-analysis raises concerns about potential biases. Lower-quality studies often lack rigorous control of confounding factors. In particular, studies that reported crude analyses without adjusting for relevant confounders may have introduced significant bias. In crude analyses from low-quality studies, an apparent association between *H. pylori* and neurodegenerative diseases may be falsely inflated or deflated due to the confounding effects of these unaccounted variables.

It is crucial to acknowledge that the observational nature of the included studies in our meta-analysis inherently limits our ability to establish causal relationships between *H. pylori* infection and neurodegenerative diseases. Although we have observed significant associations, these findings should be interpreted with caution, as they may be influenced by various factors that cannot be fully controlled in observational research. One potential concern is reverse causation. For instance, neurodegenerative diseases may alter the gut microbiota composition, which could, in turn, influence *H. pylori* colonization. Changes in gastrointestinal motility, immune function, and the production of antimicrobial peptides associated with neurodegenerative diseases might create an environment more or less conducive to *H. pylori* survival and growth. This could lead to an apparent association between *H. pylori* infection and neurodegenerative diseases, where in fact, the disease state is affecting the presence of the bacteria rather than the other way around. Residual confounding is also a significant consideration. Socioeconomic factors can play a major role in both *H. pylori* exposure and the development of neurodegenerative diseases. Lower socioeconomic status is often associated with higher rates of *H. pylori* infection due to factors such as poor sanitation, overcrowding, and limited access to healthcare. At the same time, socioeconomic factors can impact lifestyle choices, diet, and access to medical care, all of which are related to the risk of neurodegenerative diseases. Even after adjusting for some known confounders in the included studies, residual confounding from unmeasured or inadequately measured socioeconomic factors may still exist, potentially distorting the true association between *H. pylori* infection and neurodegenerative diseases.

Several limitations of this study should be acknowledged. First, as this analysis is based on cohort and case–control studies, establishing causality remains challenging. Reverse causation may be a potential issue, as neurodegenerative diseases could influence *H. pylori* colonization through alterations in the gut microbiota or other physiological changes. Second, considerable heterogeneity exists among some outcome studies, and sensitivity and subgroup analyses cannot fully account for all heterogeneity. Third, the severity of *H. pylori* infection was not considered, which may influence the strength of its association with neurodegenerative diseases. Fourth, our study is the inclusion of both adjusted and crude ORs in the primary analysis. While this approach allowed us to utilize a larger body of data, it introduced the potential for bias due to unadjusted confounding in studies reporting crude estimates. However, our subgroup analyses based on adjustment status helped to explore the impact of confounding and provided a more nuanced understanding of the associations. Future studies should aim to standardize the reporting of adjusted estimates to reduce heterogeneity and improve the accuracy of meta-analyses in this area. Fifth, the use of various *H. pylori* detection methods across studies, with their differing accuracies and potential for misclassification, represents a significant source of variability that may have affected the results of our meta-analysis. Sixth, our subgroup analyses for MS is the lack of cohort studies, the absence of cohort studies in our subgroup analyses means that we were unable to fully explore the association between *H. pylori* infection and MS from a prospective perspective. This may have influenced the stability and generalizability of our subgroup analysis results. Seventh, missing data for age and sex in some studies, indicated as ‘NA’ in Table 1, may have influenced the interpretation of our results. Although we retained these studies to maximize the sample size and noted the missing information, the lack of complete demographic data could potentially introduce bias, especially when exploring subgroup differences or the impact of confounding factors. Future research should prioritize the collection and reporting of comprehensive demographic data to improve the accuracy and reliability of meta-analyses in this field. Eighth, the impact of *H. pylori* strain virulence, such as the difference between CagA-positive and CagA-negative strains, and the treatment status of the patients were not considered in our analysis. These factors could potentially influence the association between *H. pylori* infection and neurodegenerative diseases, and may contribute to the observed heterogeneity in outcomes. However, due to the lack of stratified data on these aspects in the included studies, we were unable to explore their effects. Ninth, reliance on published literature introduces inevitable publication bias due to the inaccessibility of unpublished data. Lastly, this analysis is based on aggregated data from the included studies, limiting the depth of exploratory analyses.

This study found that *H. pylori* infection is associated with an increased risk of PD, all-cause dementia, and AD. Regarding MS, although the primary analysis showed no significant association, sensitivity analysis suggested a potential inverse association, indicating a possible “protective role” of *H. pylori*. However, this remains to be confirmed by future research. Further large-scale prospective cohort studies are necessary to validate the relationship between *H. pylori* infection and the risk of neurodegenerative diseases.

## Data Availability

The original contributions presented in the study are included in the article/Supplementary material, further inquiries can be directed to the corresponding author.
