# Supplementary material for: Association of Helicobacter pylori infection with the risk of neurodegenerative disorders: a systematic review and meta-analysis
Source: Front Med (Lausanne). 2025 Jul 4;12:1573299. doi: 10.3389/fmed.2025.1573299 (PMC12271219; doi:10.3389/fmed.2025.1573299)
Supplement: Supplementary file 1 [file Table_1.docx]

**Table S1. Search strategy in PubMed:**

| Items | Terms |
| --- | --- |
| #1 | "Neurocognitive disorders"[MeSH Terms] OR "neurological disorder*"[Title/Abstract] OR "neurodegenerative*"[Title/Abstract] OR "alzheimer*"[Title/Abstract] OR "parkinson*"[Title/Abstract] OR "huntington*"[Title/Abstract] OR "Amyotrophic lateral sclerosis"[Title/Abstract] OR "Multiple sclerosis"[Title/Abstract] OR "lewy body disease*"[Title/Abstract] OR "Frontotemporal Lobar degeneration"[Title/Abstract] OR "progressive supranuclear palsy"[Title/Abstract] OR "spinal muscular atrophy"[Title/Abstract] OR "dementia"[Title/Abstract] OR "cognitive impairment"[Title/Abstract] |
| #2 | ("helicobacter pylori"[MeSH Terms] OR ("helicobacter"[Title/Abstract] AND "pylori"[Title/Abstract]) OR "helicobacter pylori"[Title/Abstract]) |
| #3 | #1 AND #2. |


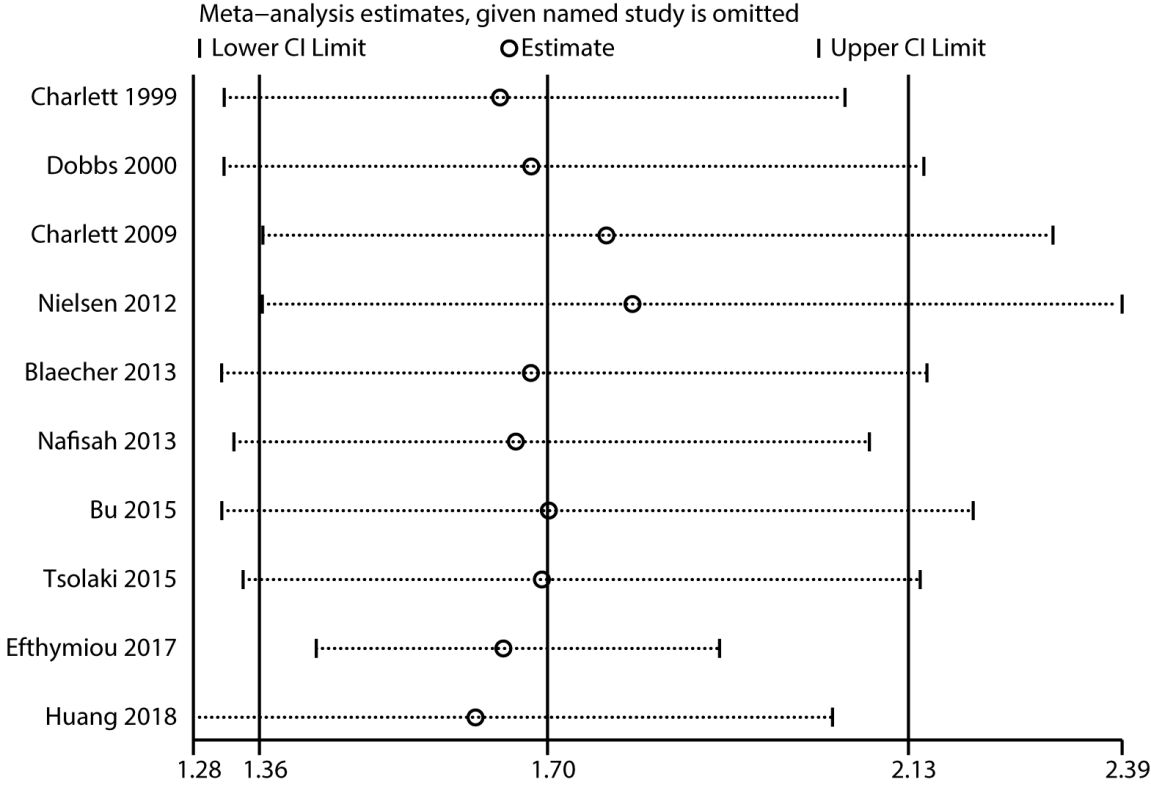


Figure S1. Sensitivity analysis for the association of *H. pylori* infection with the risk of Parkinson’s disease


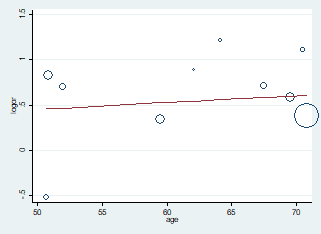


Figure S2. Meta-regression of mean age for the association between *H. pylori* infection and the risk of Parkinson’s disease (*P*=0.660).


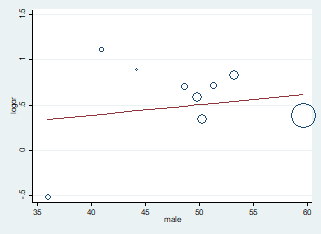


Figure S3. Meta-regression of male proportion for the association between *H. pylori* infection and the risk of Parkinson’s disease (*P*=0.592).


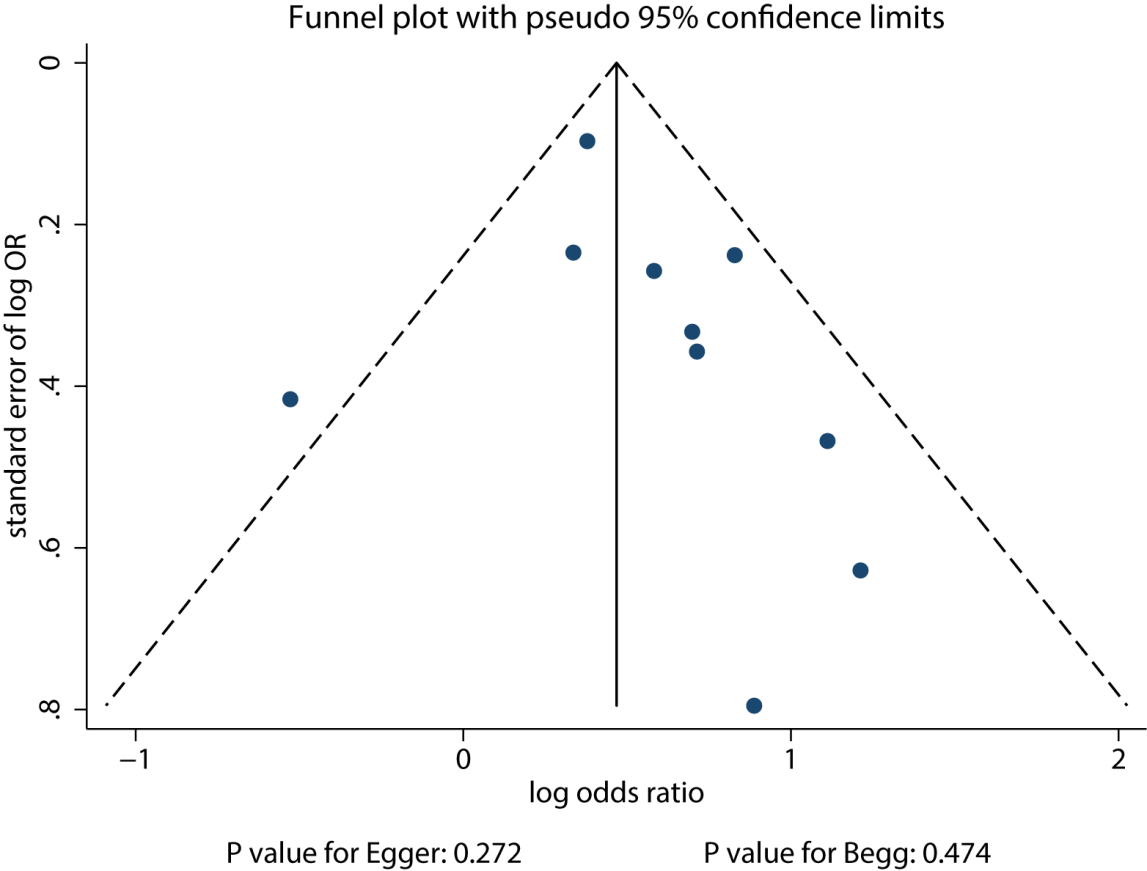


Figure S4. Funnel plot for the association of *H. pylori* infection with the risk of Parkinson’s disease


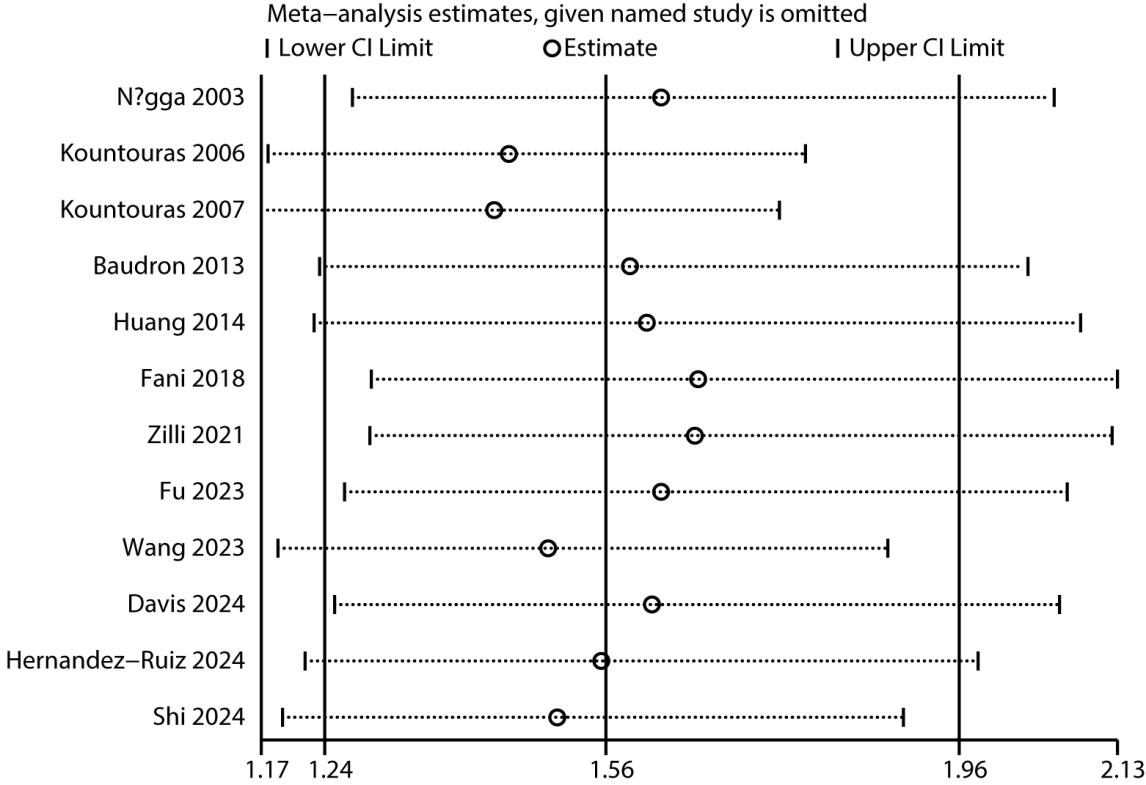


Figure S5. Sensitivity analysis for the association of *H. pylori* infection with the risk of all-cause dementia


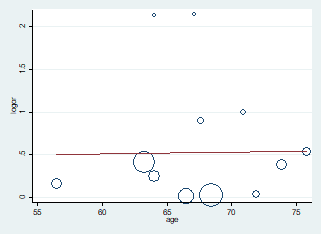


Figure S6. Meta-regression of mean age for the association between *H. pylori* infection and the risk of all-cause dementia (*P*=0.954).


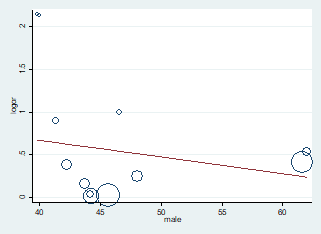


Figure S7. Meta-regression of male proportion for the association between *H. pylori* infection and the risk of all-cause dementia (*P*=0.474).


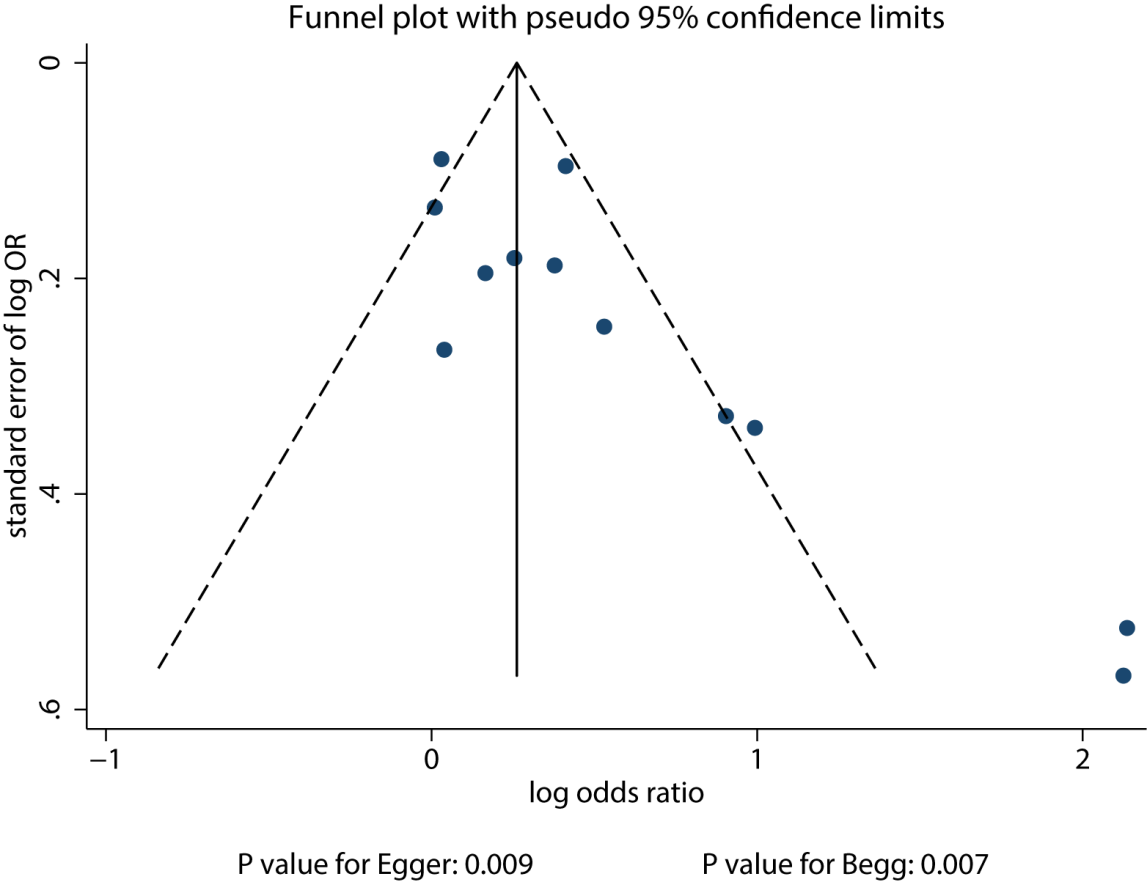


Figure S8. Funnel plot for the association of *H. pylori* infection with the risk of all-cause dementia


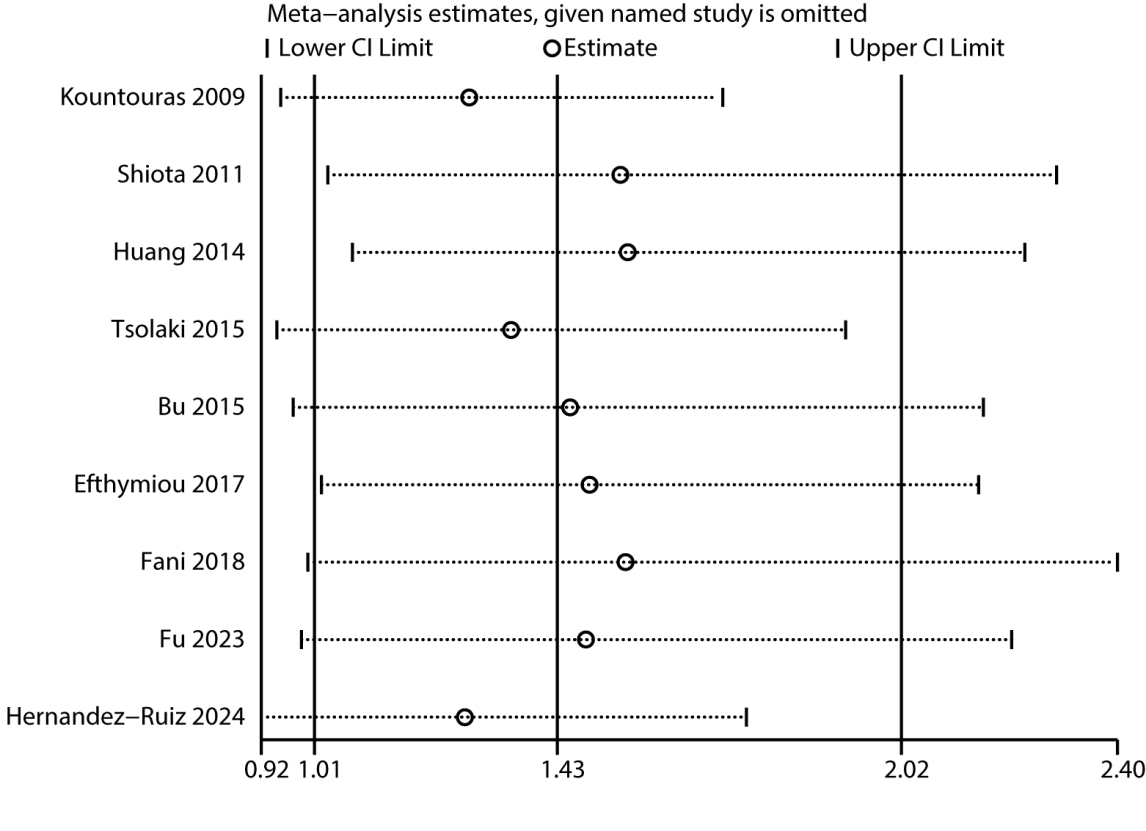


Figure S9. Sensitivity analysis for the association of *H. pylori* infection with the risk of Alzheimer’s disease


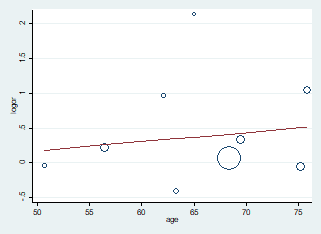


Figure S10. Meta-regression of mean age for the association between *H. pylori* infection and the risk of Alzheimer’s disease (*P*=0.687).


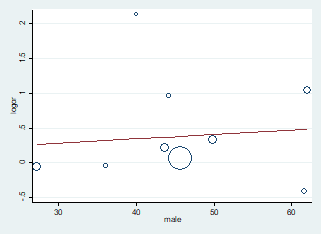


Figure S11. Meta-regression of male proportion for the association between *H. pylori* infection and the risk of Alzheimer’s disease (*P*=0.799).


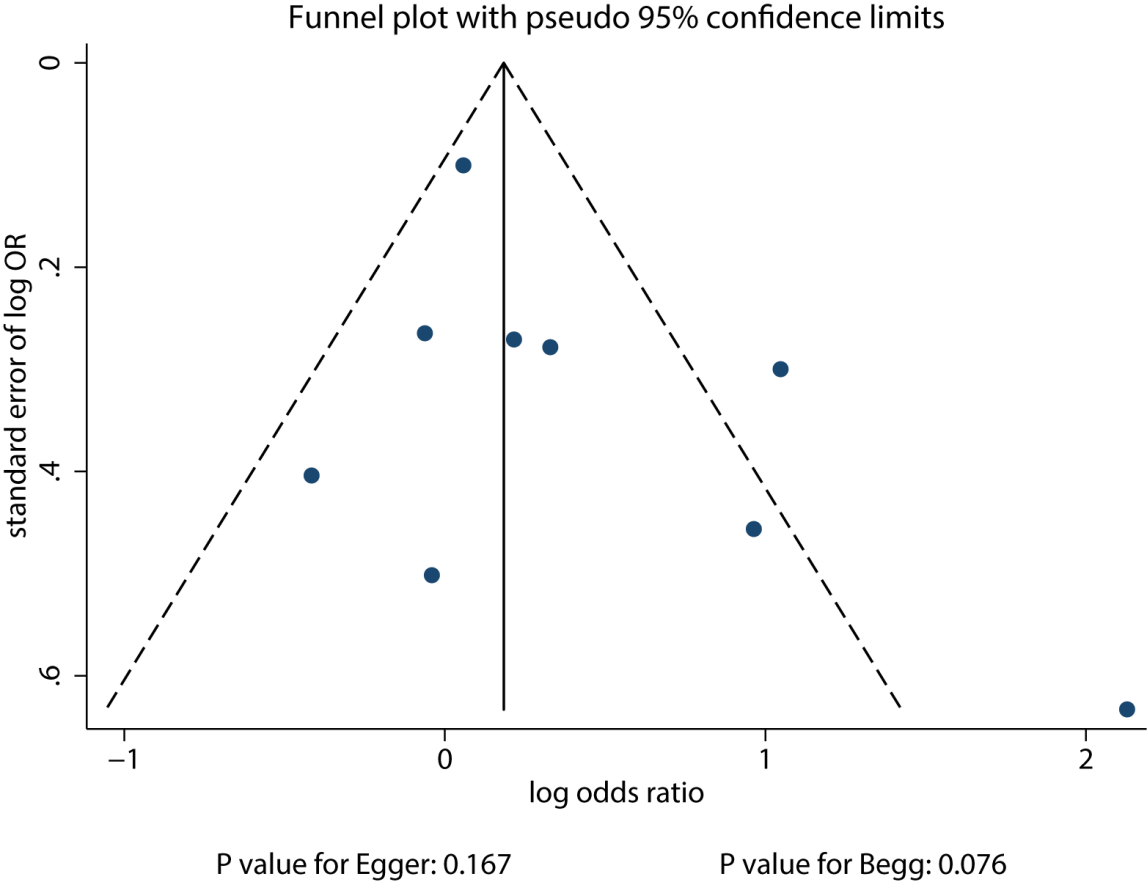


Figure S12. Funnel plot for the association of *H. pylori* infection with the risk of Alzheimer’s disease


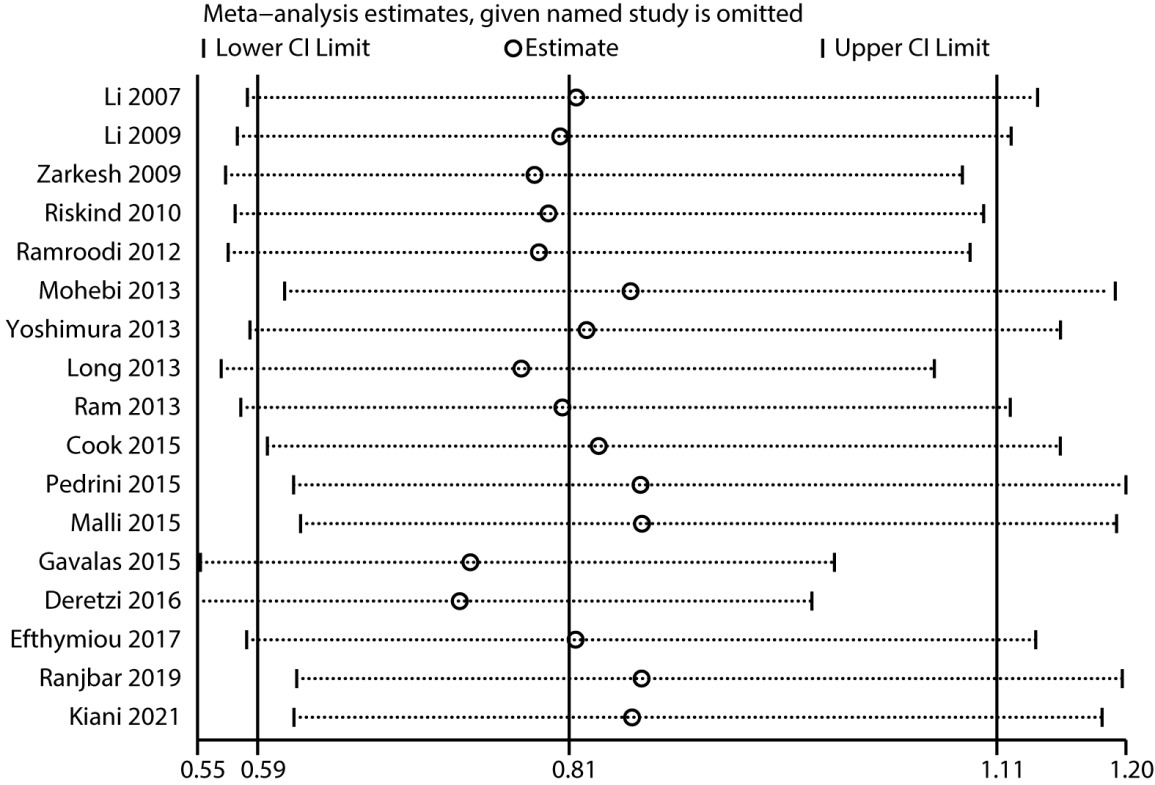


Figure S13. Sensitivity analysis for the association of *H. pylori* infection with the risk of multiple sclerosis


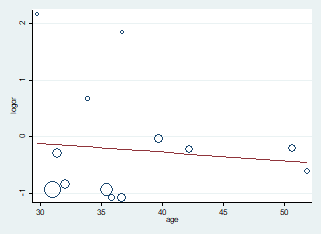


Figure S14. Meta-regression of mean age for the association between *H. pylori* infection and the risk of multiple sclerosis (*P*=0.715).


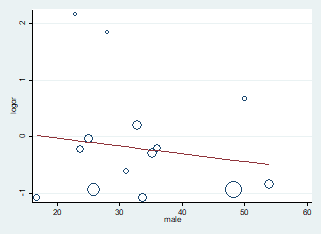


Figure S15. Meta-regression of male proportion for the association between *H. pylori* infection and the risk of multiple sclerosis (*P*=0.586).


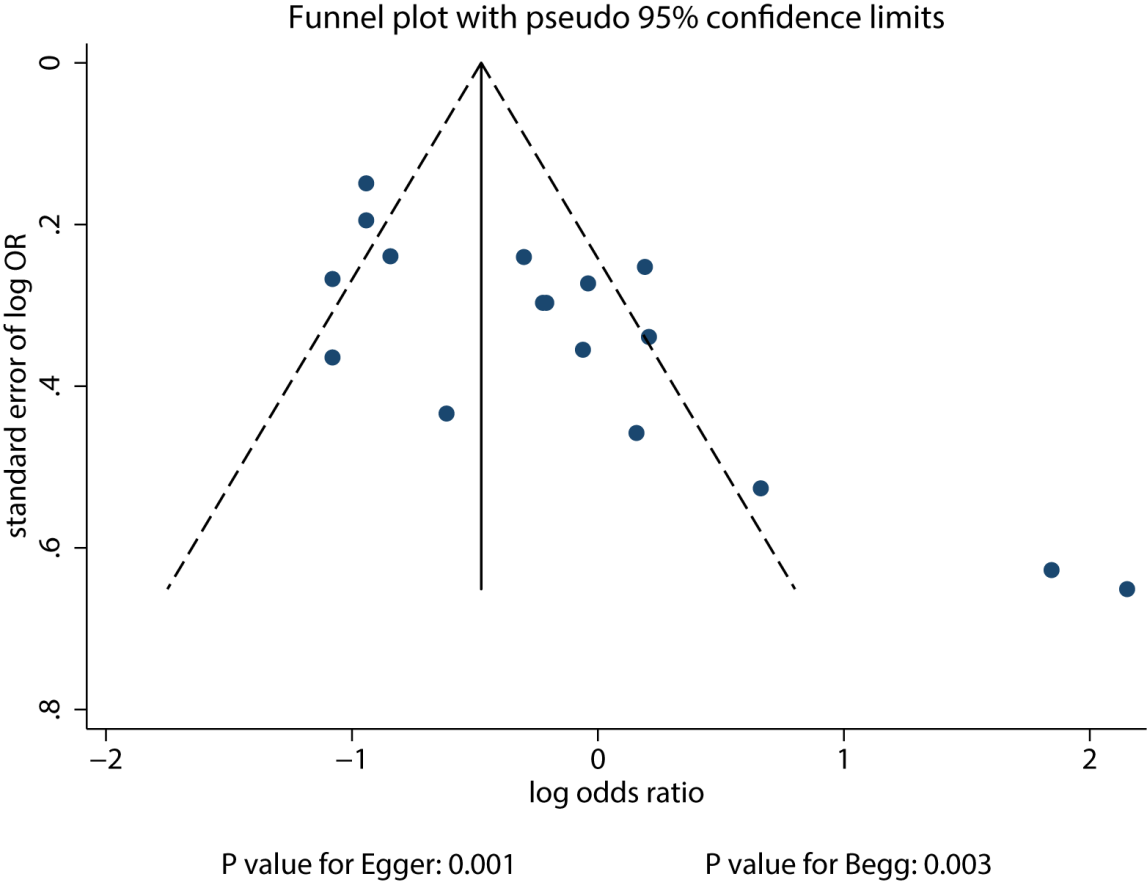


Figure S16. Funnel plot for the association of *H. pylori* infection with the risk of multiple sclerosis
